# Prediction of liquid–liquid phase separation proteins based on protein language model

**DOI:** 10.1093/bib/bbaf681

**Published:** 2025-12-17

**Authors:** Wenbin Li, Xusheng Deng, Chunlin Xiang, Hengxiang Shen, Yongyou Zhang

**Affiliations:** State Key Laboratory of Cellular Stress Biology, Innovation Center for Cell Signaling Network, Engineering Research Centre of Molecular Diagnostics of the Ministry of Education, National Institute for Data Science in Health and Medicine Engineering, School of Life Sciences, Faculty of Medicine and Life Sciences, Shenzhen Research Institute of Xiamen University, Xiamen University, No. 4221, Xiang’an South Road, Xiamen, Fujian 361102, China; State Key Laboratory of Cellular Stress Biology, Innovation Center for Cell Signaling Network, Engineering Research Centre of Molecular Diagnostics of the Ministry of Education, National Institute for Data Science in Health and Medicine Engineering, School of Life Sciences, Faculty of Medicine and Life Sciences, Shenzhen Research Institute of Xiamen University, Xiamen University, No. 4221, Xiang’an South Road, Xiamen, Fujian 361102, China; State Key Laboratory of Cellular Stress Biology, Innovation Center for Cell Signaling Network, Engineering Research Centre of Molecular Diagnostics of the Ministry of Education, National Institute for Data Science in Health and Medicine Engineering, School of Life Sciences, Faculty of Medicine and Life Sciences, Shenzhen Research Institute of Xiamen University, Xiamen University, No. 4221, Xiang’an South Road, Xiamen, Fujian 361102, China; State Key Laboratory of Cellular Stress Biology, Innovation Center for Cell Signaling Network, Engineering Research Centre of Molecular Diagnostics of the Ministry of Education, National Institute for Data Science in Health and Medicine Engineering, School of Life Sciences, Faculty of Medicine and Life Sciences, Shenzhen Research Institute of Xiamen University, Xiamen University, No. 4221, Xiang’an South Road, Xiamen, Fujian 361102, China; State Key Laboratory of Cellular Stress Biology, Innovation Center for Cell Signaling Network, Engineering Research Centre of Molecular Diagnostics of the Ministry of Education, National Institute for Data Science in Health and Medicine Engineering, School of Life Sciences, Faculty of Medicine and Life Sciences, Shenzhen Research Institute of Xiamen University, Xiamen University, No. 4221, Xiang’an South Road, Xiamen, Fujian 361102, China

**Keywords:** liquid–liquid phase separation (LLPS), protein language model (PLM), machine learning, deep learning, feature engineering

## Abstract

Liquid–liquid phase separation (LLPS) enables biomolecules to form membraneless condensates critical for cellular functions like RNA metabolism and protein synthesis. Identifying LLPS-associated proteins is essential for understanding their roles in cellular organization and disease. Current prediction methods are hindered by complex and inefficient feature extraction processes. Here, we present a novel framework combining a protein language model ProtT5 with a KmerConv module for local sequence pattern detection and a multi-head attention mechanism for global sequence information extraction. This integrated approach achieves high predictive performance across multiple diverse datasets and generalizes effectively across different species. Our method provides a robust and efficient tool for systematic LLPS protein identification, advancing research into biomolecular aggregation and its implications for health and disease.

## Introduction

Intracellular liquid–liquid phase separation (LLPS) involves the aggregation of biomolecules [1], such as proteins and nucleic acids, within cells to form organized structures known as biomolecular liquid condensates [2]. These condensates create membraneless organelles [3], like nucleoli, stress granules, and P-bodies, which play vital roles in biological processes (BPs), including RNA metabolism, protein synthesis, and signal transduction. By concentrating signaling molecules [4], LLPS provides spatial and temporal flexibility, enhancing the efficiency of biochemical reactions and cellular functions. Furthermore, phase separation influences developmental signaling [5], chromatin organization, and gene expression [6], with transcription factors and epigenetic enzymes modulating chromatin regions [7, 8]. Phase-separating proteins (PSPs) often contain disordered regions or specific motifs, such as leucine and arginine-rich sequences [9], which facilitate the formation of these condensates. Under stress conditions, phase separation forms protective structures like stress granules, helping cells recover by safeguarding RNA and proteins from damage [10, 11]. Recent studies have linked LLPS to neurodegenerative diseases like Alzheimer’s disease [12] and Parkinson’s disease [13], as well as cancers [14, 15, 16, 17]. As drug discovery becomes more complex, targeting phase separation and related pathways, such as lysosome/exosome processes, shows promise for developing new therapies, including antiviral strategies [18]. Thus, understanding PSPs is crucial for exploring their biological significance and potential therapeutic applications.

Many methods for predicting PSPs have been developed, including tools like PScore [19], which is based on Pi–Pi concat interactions, PLAAC [20] for identifying segments rich in ketone amino acids, and catGRANULE [21], which combines RNA-binding and disorder propensity. Other models, such as PSAP [22], use amino acid properties for prediction, while imaging-based validation methods, like DeepPhase [23], assess droplet-like structures. These models often focus on specific aspects of phase separation, providing insights from a single perspective. In contrast, PhaSePred [24] combines multiple prediction models and is the first to identify self-assembly and chaperone-dependent PSPs. Recent advances, such as the PSPire [25], improve prediction accuracy by incorporating structural information for proteins lacking intrinsically disordered regions (IDRs). Additionally, the PSPHunter [26] integrates sequence, evolutionary, and functional data to predict key residues in PSPs.

Traditional PSP prediction methods typically focus on specific sequence features and require additional tools, such as GPSFun [27], to explore post-translational modifications (PTMs) and evolutionary information. However, this feature engineering process can be time-consuming and may miss certain protein properties. Deep learning offers a powerful alternative by automatically learning and extracting valuable features from raw data, reducing the need for expert knowledge and explicit feature selection [28]. This end-to-end learning approach improves model performance and adaptability to new data. In the era of big data, large-scale protein datasets can be used to train models like ProtTrans, a protein language model (PLM) based on the Transformer architecture [29]. By capturing biophysical characteristics within protein sequences, it has shown strong effectiveness in understanding amino acid sequence patterns, as demonstrated by various studies [30, 31, 32].

To ensure the reliability of negative samples, we applied a two-step filtering strategy based on sequence similarity and imaging validation. Leveraging ProtT5, we designed a dual-stream architecture that couples KmerConv-based 1-D convolutions for local motif extraction with Multi-Head Attention (MHA) for global context integration. This method simplifies feature engineering while achieving superior performance compared to existing models. Extensive experiments on independent datasets confirmed the robustness of the approach. We further conducted an exploratory analysis of putative proteins, clustered them according to interaction networks, and corroborated our findings by integrating microscopy images and recent literature, underscoring the method’s potential to advance LLPS research.

## Materials and methods

### Data collection and collation

To comprehensively collect data, we gathered information from several databases, including PhaSepDB [33], DrLLPS [34], LLPSDB [35], and PhaSePro [36], as well as recent studies [25, 26]. In total, we collected 958 PSPs, consisting of 640 human LLPS proteins and 318 non-human LLPS proteins. For human negative samples, after using CD-Hit [37] with a 0.3 threshold to remove redundant sequences, which retained 5754 samples. To minimize the inclusion of potential LLPS proteins in the negative samples, we validated the proteins using the subcellular immunofluorescence image database HPA [38], which includes images from three cell lines: U2-OS, U-251MG, and A-431. We examined 1338 proteins with images available for these three cell lines, and 717 proteins displayed a fully diffuse distribution, which we considered reliable negative samples.

The fluorescence images from the HPA database consist of four-channel images: red, green, blue, and yellow. For our analysis, we utilized the red and green channels, where the red channel highlights the cytoskeleton (cell fibers) and the green channel represents the target protein. Images from U2-OS, U-251MG, and A-431 cell lines were selected due to their abundance and suitability for validation. For non-human negative samples, we curated a dataset comprising a total of 39 358 entries across various species, with the detailed breakdown provided in Table 1.

**Table 1:** Non-human negative sample statistics.

**Species**	**Count**
*Mus musculus*	9746
*Arabidopsis thaliana*	6392
*Rattus norvegicus*	4875
*Saccharomyces cerevisiae*	4554
*Schizosaccharomyces pombe*	3707
*Caenorhabditis elegans*	2874
*Escherichia coli*	2803
*Drosophila melanogaster*	2494
*Xenopus laevis*	1899

### Dataset construction

We divided the dataset into a 7:3 ratio for the TrainDataSet and TestDataSet, with the negative samples being proteins without droplet tendencies as determined by image filtering (Supplementary Material S1). To test the model’s generalization ability, we constructed three independent test datasets for validation. These include the construction of a non-human LLPS protein validation set, Non-humanDataSet (Supplementary Material S4). Since this set does not have protein fluorescence images for filtering, we randomly selected 494 negative samples and combined them with all 318 positive samples to form the dataset. Additionally, two MLO-related datasets, G3BP1-DataSet [39] and RNAGrunaleDB-DataSet [40] (Supplementary Material S3), were used. The G3BP1 proximity labeling set mainly consists of proteins with relative abundance greater than 0.01 compared to the G3BP1 protein. RNAgranuleDB primarily consists of stress granules and P-bodies. Both datasets are highly imbalanced. In the G3BP1 DataSet, there are a total of 1968 samples, with 1810 negative samples and 158 positive samples. In RNAgranuleDB, there are 2040 samples, with 222 positive samples and 1818 negative samples. To prevent self-validation, proteins included in both the training and test datasets were removed.

### Protein language model validation strategy

To validate the effectiveness of the PLM for protein embedding encoding, we first applied the same data as used in PhaSePred and PSPire for validation. For PhaSePred, we curated 87 human self-assembling LLPS proteins (hSaPSPs), 149 human partner-dependent LLPS proteins (hPdPSPs), and human non-LLPS proteins (hNoPSPs). The training and test sets were divided according to the method used in PhaSePred, where we randomly selected two times the number of positive samples from non-LLPS proteins to build the training dataset, and the same proportion of negative test samples as the positive test data to form the test set. For PSPire, we curated 128 non-IDR-driven LLPS proteins (woIDR-PSPs), 389 IDR-driven LLPS proteins (wIDR-PSPs), and 10 284 non-LLPS proteins. Positive samples for training and testing were split in a 1:1 ratio, while the negative samples used for training included 8323 data points, and 1961 data points were used for test validation.

### Protein language model embedding

Currently, PLMs are widely used in the fields of bioinformatics and computational biology. These models primarily utilize the Transformer architecture from the NLP domain, which has been trained on vast datasets, treating protein sequences as text and amino acids as words. Several studies have applied large language models to learn patterns within protein sequences, and the learned embeddings are then used for protein-level tasks, such as secondary structure prediction [41] and protein function prediction [27]. In this study, we used the ProtT5-UniRef50 [29] architecture’s Encoder and ESM2(esm2_t33_650M_UR50D) [42] to extract embedding features for each residue. Protein sequences were represented as position-wise embedding matrices, with dimensional specifics varying by language model: ProtT5 generates L × 1024 matrices versus ESM2’s L × 1280 matrices (where L = sequence length).

### Machine learning model building

In this work, we evaluated four machine learning models, including Support Vector Machine (SVM) [43], Random Forest [44], eXtreme Gradient Boosting [45], and Light Gradient Boosting Machine [46]. We used the sklearn package, the Xgboost package, and the Lightgbm package in Python to build the algorithm models. In the implementation process, the SVM was set with probability = True for probabilistic output, and the rest of the parameters were kept at their default settings.

### Deep learning model building

We propose a new framework that combines a PLM and a KmerConvEncoder dual model. As shown in the Fig. 2B, after extracting local features of the protein sequence through the KmerConv module, the global information is integrated and understood from multiple perspectives through the MHA mechanism. The one-dimensional convolution is implemented using a sliding window, where this window moves across the input sequence, and each slide computes the sum of the product of the elements in the window and the elements in the convolution kernel. Given an input sequence *I* = [${I}_0$​,${I}_1$,…, ${I}_{N-1}$] of length *N* and a convolution kernel *K* = [${K}_0$​,${K}_1$​,…, ${K}_{m-1}$​] of length *m*, the convolution result is an output sequence *C* = [​${C}_0$, ${C}_1$ ​,…, ${C}_{n-m}$ ​] of length *N − m + 1*, where each element is calculated as:


(1) \begin{equation*} {C}_n=\sum_{k=0}^{m-1}{I}_{n+k}\cdotp{K}_{m-1-k},n=0,1,\dots, N-m \end{equation*}

In the above formula, the convolution kernel *K* is flipped before being multiplied element-wise with the input sequence *I* and summed to obtain the n-th element of the output sequence *C*.

The MHA mechanism is based on the self-attention mechanism and is widely used in natural language processing tasks. For each head, the *Q*, *K*, and *V* matrices are obtained by applying the corresponding weight matrices ${W}_{Qi}$, ${W}_{Ki}$, and ${W}_{Vi}$ to the input *X* through linear transformations, as shown in the following formula:


(2) \begin{equation*} {\displaystyle \begin{array}{c}{Q}_i=X{W}_{Qi}\end{array}} \end{equation*}

(3) \begin{equation*} {\displaystyle \begin{array}{c}{K}_i=X{W}_{Ki}\end{array}} \end{equation*}

(4) \begin{equation*} {\displaystyle \begin{array}{c}{V}_i=X{W}_{Vi}\end{array}} \end{equation*}

For each head, the dot product of *Q* and *K* is computed and converted to attention weights via the *softmax* function, with the scaling factor $\sqrt{d_k}$ (where ${d}_k$is the dimension of the key), as shown in the following formula:


(5) \begin{equation*} {\displaystyle \begin{array}{c}{A}_i=\mathrm{softmax}\left(\frac{Q_i{K}_i^T}{\sqrt{d_k}}\right)\end{array}} \end{equation*}

For each head, a weighted sum of the values is performed using the attention weights to obtain the output of each head. The formula is as follows:


(6) \begin{equation*} {\displaystyle \begin{array}{c}{O}_i={A}_i{V}_i\end{array}} \end{equation*}

The final output is obtained by concatenating the outputs of all heads and applying a linear transformation with the output weight matrix ${W}_O$, as shown in the following formula:


(7) \begin{equation*} {\displaystyle \begin{array}{c}\mathrm{MultiHead}\left(Q,K,V\right)=\mathrm{Concat}\left({O}_1,{O}_2,\dots, {O}_h\right){W}_O\end{array}} \end{equation*}

The combined vector ${x}_m$ is formed by concatenating the ProtT5-encoded vector ${x}_{protT5}$ (dimension ${d}_1$) with the KmerConvEncoder-encoded vector ${x}_{KmerConv}$ (dimension ${d}_2$), yielding dimension ${d}_1+{d}_2$, as shown in the following formula:


(8) \begin{equation*} {\displaystyle \begin{array}{c}{x}_{\mathrm{m}}=\mathrm{Concatenate}\left({x}_{protT5},{x}_{KmerConv}\right)\end{array}} \end{equation*}

The decoder transforms the input $x$ via a stack of fully-connected, activation and normalization layers parameterized by weights ${W}_1$, ${W}_2$ and ${W}_3$ as expressed below:


(9) \begin{equation*} {\displaystyle \begin{array}{c}{x}_1=\mathrm{Dropout}\left(\mathrm{LayerNorm}\left(\mathrm{Tanh}\left({W}_1x\right)\right)\right)\end{array}} \end{equation*}

(10) \begin{equation*} {\displaystyle \begin{array}{c}{x}_2=\mathrm{Dropout}\left(\mathrm{LayerNorm}\left(\mathrm{LeakyReLU}\left({W}_2{x}_1\right)\right)\right)\end{array}} \end{equation*}

(11) \begin{equation*} {\displaystyle \begin{array}{c}{x}_3=\mathrm{Dropout}\left({W}_3{x}_2\right)\end{array}} \end{equation*}

The learning rate adjustment strategy uses WarmupLR, where the learning rate is scaled by the ratio of the current step number (*num_step*) to the warm-up step count (*num_warm*). The formula is as follows:


(12) \begin{equation*} {\displaystyle \begin{array}{c}\mathrm{lr}=\mathit{\min}\left(\mathrm{lr}\times \mathrm{num}\_{\mathrm{step}}^{-0.6},\mathrm{lr}\times \mathrm{num}\_\mathrm{step}\times \mathrm{num}\_{\mathrm{warm}}^{-1.5}\right)\end{array}} \end{equation*}

The entire model framework is implemented based on Pytorch, we trained and tested it on NVIDIA GeForce RTX 3090 (cuda12.4 environment), using Adam optimizer. The training times are 50 rounds, and the Kmer size is set to 5. To evaluate the model robustness, we performed five-fold cross-validation on the training data for the primary model, with the random seed set to 2024.

### Evaluation

To comprehensively evaluate the performance of the model, the following metrics are used: Accuracy (ACC), Precision (PRE), Recall (REC), F1 Score (F1), Matthews correlation coefficient (MCC), area under the receiver operating characteristic curve (AUROC), and area under the precision-recall curve (PRAUC). The formulas are as follows:


(13) \begin{equation*} \mathrm{PRE}=\frac{TP}{TP+ FP} \end{equation*}

(14) \begin{equation*} \mathrm{REC}=\frac{TP}{TP+ FN} \end{equation*}

(15) \begin{equation*} F1=2\cdotp \frac{PRE\cdotp REC}{PRE+ REC} \end{equation*}

(16) \begin{equation*} \mathrm{ACC}=\frac{TP+ TN}{TP+ TN+ FP+ FN} \end{equation*}

(17) \begin{equation*} MCC=\frac{TP\cdotp TN- FP\cdotp FN}{\sqrt{\left( TP+ FP\right)\left( TP+ FN\right)\left( TN+ FP\right)\left( TN+ FN\right)}} \end{equation*}

Where *TP, FP, TN,* and *FN* represent True Positives, False Positives, True Negatives, and False Negatives, respectively.

### Enrichment analysis

To comprehensively understand the distribution characteristics of positive samples across the entire human proteome, we performed Gene Set Enrichment Analysis (GSEA). GSEA is a bioinformatics method used to determine whether a predefined gene set shows significant enrichment trends within a larger gene set. In this study, we used the gseapy package in Python [47] to perform GSEA enrichment analysis and examine the distribution of samples across the entire proteome, thereby validating the reliability of the model.

### Protein analysis

To further investigate these candidate proteins, we utilized the STRING database [48] for analysis. The STRING database allows for clustering analysis based on protein–protein interaction information (PPI), which includes both direct physical contacts and indirect interactions mediated by other molecules. In this process, proteins with stronger interaction intensities are grouped together to form a cluster, while those with weaker or no significant interactions are assigned to different clusters. This approach enables STRING to reveal the functional networks of proteins within cells, helping us understand the complex interactions in BPs.

In our study, we used the K-Means Clustering method for the clustering analysis and employed Cytoscape [49] for visualization. Additionally, we performed Gene Ontology (GO) enrichment analysis and KEGG pathway analysis to further understand the functions of these proteins and their roles in BPs. All analyses were conducted using default parameter settings.

## Results and discussion

### Validation of protein language model

To evaluate the effectiveness of PLMs in identifying LLPS proteins, we employed four machine learning algorithms—SVMs [43], Random Forest [44], XGBoost [45], and LightGBM [46]—to construct models, with Receiver Operating Area Under the Curve (ROAUC) as the primary evaluation metric. Additionally, we systematically compared two PLMs, ProtT5 and ESM2, across diverse computational tasks.

**Figure 1:**
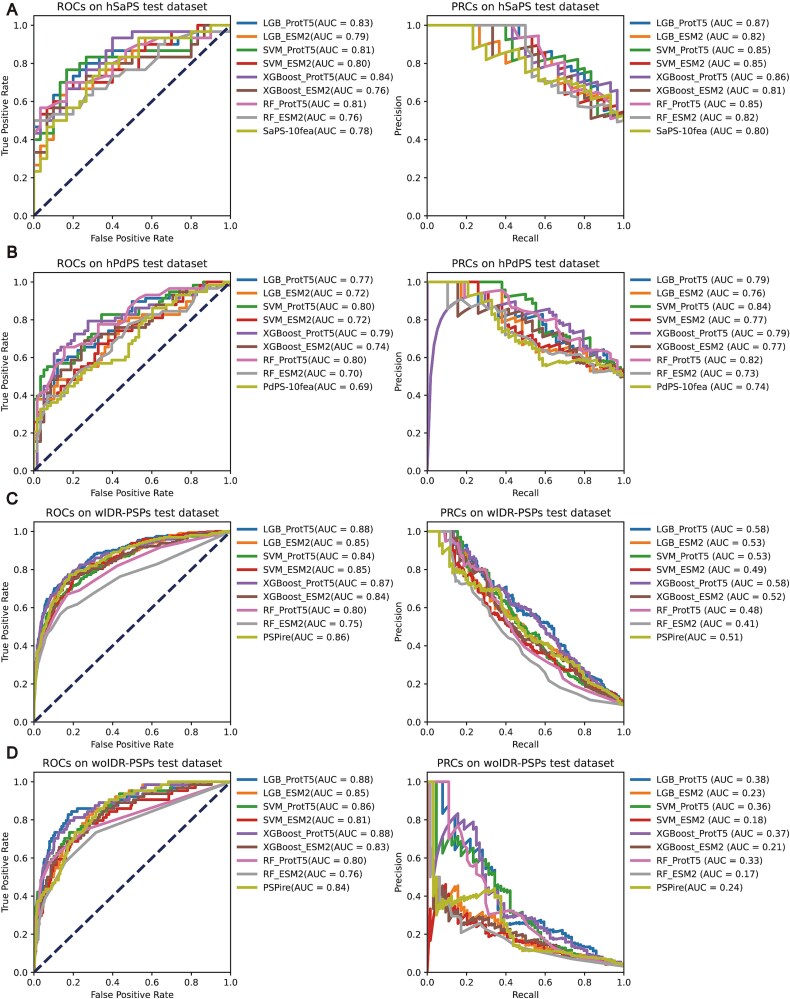
Performance comparison of ProtT5- and ESM2-based protein representations across four downstream tasks, benchmarked against original model architectures. (A) ROAUC and PRAUC performance metrics on hSaPS (human self-assembling phase separation proteins) test datasets. (B) ROAUC and PRAUC performance metrics on hPdPS (human partner-dependent phase separation proteins) test datasets. (C) ROAUC and PRAUC performance metrics on wIDR-PSPs (phase-separating proteins with IDR) test datasets. (D) ROAUC and PRAUC performance metrics on woIDR-PSPs test datasets (phase-separating proteins without IDR).

PhaSePred [24] was the pioneering model developed to screen self-assembly and partner-dependent PSPs. In self-assembling protein classification (hSaPS test dataset), models utilizing ProtT5 feature encoding demonstrated significantly superior performance, achieving ROAUC values >0.8 and PRAUC >0.85. In contrast, ESM2-based models exhibited performance fluctuations near baseline levels (ROAUC = 0.78, PRAUC = 0.8), consistently underperforming relative to ProtT5 (Fig. 1A). Similarly, for partner-dependent protein identification (hPdPS test dataset), ProtT5 encoding again showed enhanced predictive capability (ROAUC >0.77 vs. baseline 0.69; PRAUC >0.79 vs. baseline 0.74), whereas ESM2-based models continued to show suboptimal predictive performance (Fig. 1B).

PSPire [25] integrates structural information (SSUP) for predicting PSPs mediated by non-IDRs. In the prediction of IDR-mediated phase separation (wIDR-PSPs test dataset), ProtT5-enabled models attained ROAUC values >0.8, with the LightGBM algorithm achieving particularly strong performance (ROAUC = 0.88), representing a 0.02 improvement over the PSPire baseline (ROAUC = 0.86). ESM2-based models reached a maximum ROAUC of 0.85 in this task (Fig. 1C). Notably, for IDR-independent phase separation prediction (woIDR-PSPs test dataset), ProtT5-based models again outperformed ESM2, with LightGBM and XGBoost implementations achieving ROAUC values of 0.88 compared to the PSPire baseline of 0.84 (Fig. 1D).

Our results demonstrate that models utilizing PLM-based feature encoding consistently outperform baseline techniques across all evaluated tasks. In particularly, ProtT5-based models surpassed those using ESM2, achieving higher ROAUC and PRAUC scores in self-assembling protein classifying, partner-dependent protein identification, and both IDR-mediated and IDR-independent phase separation prediction. These findings highlight ProtT5’s effectiveness as a robust feature encoding strategy for protein-related machine learning tasks and underscore the performance disparities among different PLMs.

### Model building

**Figure 2:**
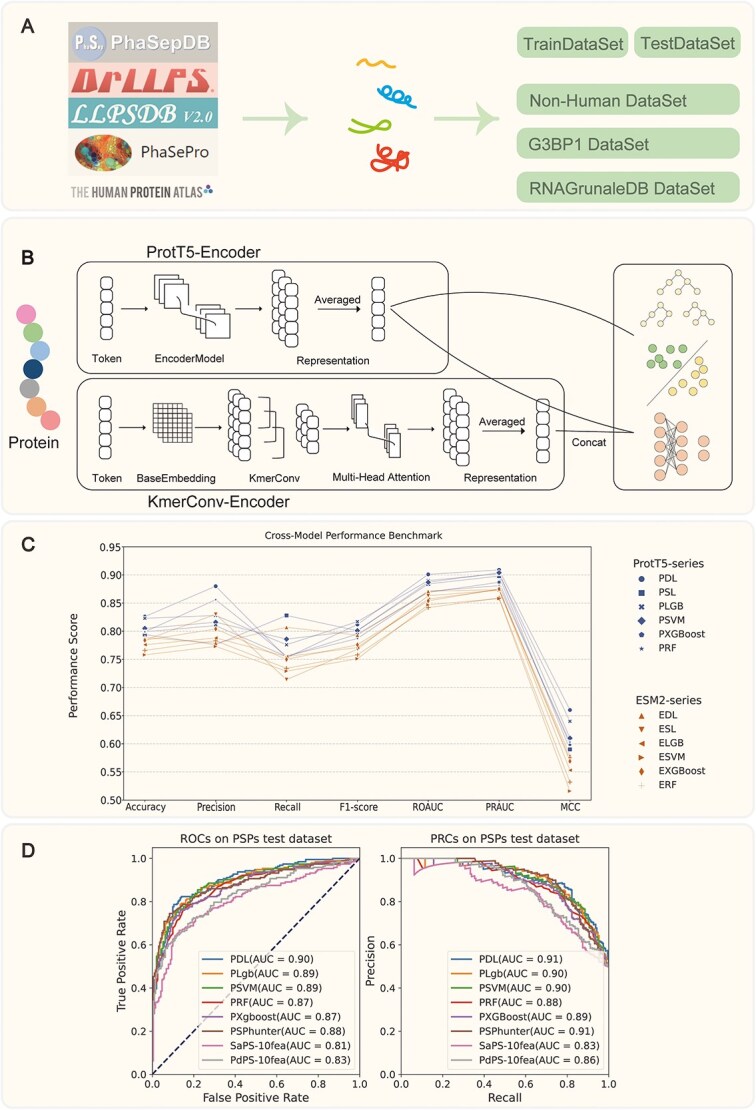
The framework of our research. (A) Data collection and integration process. (B) Method and technology roadmap. The three diagrams in the right panel demonstrate distinct computational frameworks: The upper diagram illustrates ensemble architectures (Random Forest, XGBoost, LightGBM), the central schematic demonstrates SVM-based hyperplane feature space partitioning, and the lower visualization implements a linear decoder architecture. (C) Comparison of models based on ProtT5 and ESM2. (Model nomenclature: P-prefixed identifiers denote ProtT5-based architectures, while E-prefixed codes indicate ESM2-derived frameworks. DL represents our dual-model configuration, SL corresponds to single-PLM architecture, with remaining abbreviations designating comparative machine learning baselines. (D) Comparison of our method with existing methods.

We collected and organized a set of training and testing data based on existing phase-separation protein databases and related literature. To filter out potential negative PSPs, we removed redundant single-structure proteins and validated protein images from the subcellular immunofluorescence image database HPA [38]. This process involved eliminating proteins with slight droplet or aggregation tendencies, further ensuring the validity of negative samples (Fig. 2A).

We designed a novel hybrid architecture by integrating two computational modules: (i) a KmerConv component for local sequence pattern extraction and (ii) an MHA mechanism for global feature integration. These components were combined to form a KmerConv-Encoder module, which was then incorporated into the established ProtT5Encoder framework, creating an enhanced dual-model architecture for protein sequence analysis (Fig. 2B). The KmerConv module employs 1D convolution to extract local sequence features, capturing microstructural patterns similar to edge detection in image processing [50]. In contrast, the MHA module dynamically weights these local features, integrating them into a global representation while preserving local details [51]. This integration enables a holistic understanding of the protein sequence while preserving local details.

### Benchmarking against existing predictors

We systematically evaluated two PLMs (ProtT5 and ESM2) integrated with distinct computational architectures. The ProtT5-based framework incorporated three configurations: (i) a dual-language model (PDL), (ii) a single-protein baseline (PSL), and (iii) supervised learning implementations [lightGBM (PLGB), SVM (PSVM), XGBoost (PXGB), and Random Forest (PRF)]. A parallel ESM2-based framework was implemented with equivalent components, including a dual-language model (EDL), single-protein baseline (ESL), and corresponding machine learning variants (ELGB, ESVM, EXGB, and ERF).

The ProtT5-based models consistently outperformed their ESM2 counterparts across all evaluation metrics (Fig. 2C). Notably, the dual-architecture ProtT5 model (PDL) achieved state-of-the-art performance (detailed comparisons in Table 2), with classification Accuracy (0.826), ROAUC (0.901), PRAUC (0.909), and MCC (0.660). These results highlight the effectiveness of ProtT5’s hierarchical feature fusion mechanism in capturing multi-scale protein interaction patterns.

**Table 2:** Comparison results of each model on test dataset.

Model	Accuracy	Precision	Recall	F1-score	ROAUC	PRAUC	MCC
**PDL**	**0.826**	**0.880**	0.753	0.812	**0.901**	**0.909**	**0.660**
PSL	0.794	0.776	0.828	0.801	0.884	0.898	0.590
PLGB	0.823	0.828	0.776	**0.817**	0.890	0.902	0.647
PSVM	0.805	0.816	0.786	0.801	0.887	0.904	0.610
PXGBoost	0.799	0.810	0.755	0.795	0.869	0.887	0.599
PRF	0.799	0.856	0.756	0.787	0.871	0.881	0.603
EDL	0.789	0.779	0.807	0.793	0.871	0.874	0.579
ESL	0.784	0.830	0.714	0.768	0.863	0.873	0.573
ELGB	0.776	0.788	0.755	0.771	0.857	0.874	0.553
ESVM	0.758	0.773	0.729	0.751	0.847	0.858	0.516
EXGBoost	0.784	0.804	0.750	0.776	0.854	0.875	0.569
ERF	0.766	0.783	0.734	0.758	0.842	0.859	0.532
PSPHunter	0.805	0.794	0.823	0.808	0.879	0.905	0.610
PdPS	0.672	0.623	**0.869**	0.726	0.831	0.862	0.374
SaPS	0.701	0.661	0.823	0.733	0.807	0.833	0.414

Further analysis against advanced predictors (PSPHunter, SaPS, and PdPS) demonstrated the robustness of our framework (Fig. 2D). While PSPHunter exhibited competitive performance in ROAUC (0.88 vs. 0.90 for PDL) and equivalent PRAUC (0.901), our ProtT5-based model maintained superior Accuracy (0.826 vs. 0.806), Precision (0.880 vs. 0.800), and MCC (0.660 vs. 0.610). This performance advantage highlights the effectiveness of our architecture in capturing and integrating both local and global sequence features, thereby enhancing predictive accuracy across diverse phase separation contexts. Meanwhile, Cross-validation results (Supplementary Material Table 1) show that PDL achieved ≤5% RSD across all primary metrics (F1-score: 76.2% ± 2.1%, ROC-AUC: 86.9% ± 3.3%, PRAUC: 86.8% ± 2.1%), with ROC-AUC’s 3.8% RSD confirming method stability under varying data splits. Moreover, taken as a whole, the ProtT5-based models outperform their ESM2 counterparts on the majority of evaluation metrics.

### Independent sample verification

To further validate the model’s generalization ability, we conducted testing on independent samples, including two human MLO participant datasets: the G3BP1 proximity labeling set [39] and RNAgranuleDB [40], as well as a non-human PSP dataset. As shown in Table 3, our model outperformed PSPHunter in terms of overall performance metrics on these two datasets. Specifically, on the G3BP1 dataset, the ROAUC reached 0.932 and the PRAUC reached 0.804, which is approximately 0.2 higher than PSPHunter. On the RNAgranuleDB dataset, the ROAUC was 0.866 and the PRAUC was 0.590, with all other performance metrics exceeding those of the other models.

**Table 3:** Comparison results of each model on independent dataset.

IndDataSet	Model	Accuracy	F1-score	ROAUC	PRAUC	MCC
G3BP1	PDL	**0.837**	**0.479**	0.932	**0.804**	**0.491**
	PSPHunter	0.822	0.453	**0.941**	0.610	0.462
	SaPS	0.593	0.264	0.842	0.290	0.259
	PdPS	0.601	0.274	0.850	0.351	0.277
RNAgranuleDB	PDL	**0.818**	**0.478**	**0.866**	**0.590**	**0.430**
	PSPHunter	0.805	0.450	0.864	0.536	0.396
	SaPS	0.583	0.282	0.742	0.350	0.196
	PdPS	0.598	0.310	0.786	0.350	0.196
Non-human	PDL	0.711	0.622	0.800	0.727	0.417
	PSPHunter	**0.716**	**0.694**	**0.818**	**0.734**	**0.460**

**Figure 3:**
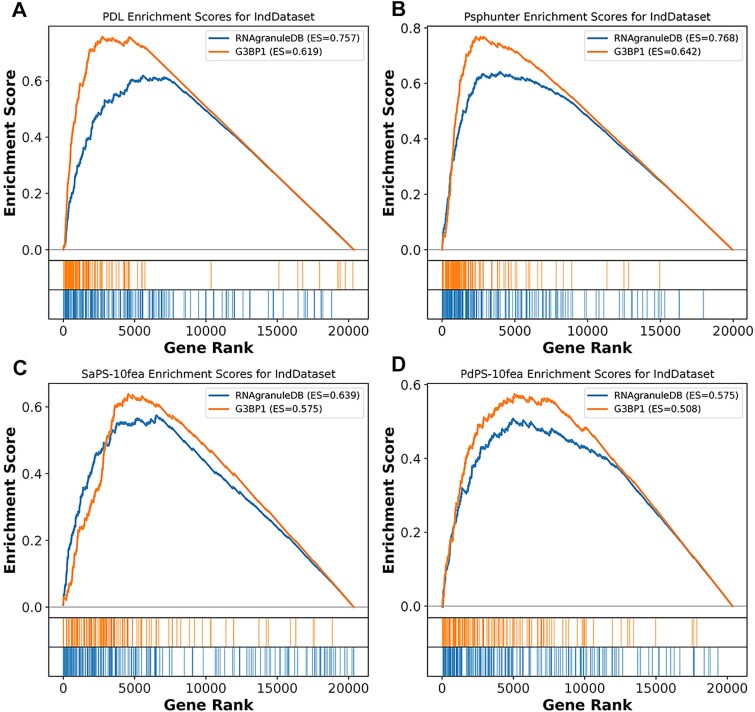
Enrichment analysis on independent sample test data. (A) PDL-Model Enrichment Scores for IndDataset. (B) PSPHunter Enrichment Scores for IndDataset. (C) SaPS-Model Enrichment Scores for IndDataset. (D) PdPS-model Enrichment Scores for IndDAtaset.

To assess the model’s species transferability, we also validated it on the non-human PSP dataset. The F1-Score reached 0.662 and the ROAUC was 0.8, showing robust transferability, albeit slightly lower than PSPHunter. This is likely due to the model being trained primarily on human proteins, and the differences in protein characteristics across species. Additionally, we performed an enrichment ranking for positive samples across the entire proteome, as shown in Fig. 3. The positive samples were ranked toward the top, with enrichment scores of 0.757 and 0.619 on the two independent test datasets, respectively.

### Feature dimensionality reduction visualization

**Figure 4:**
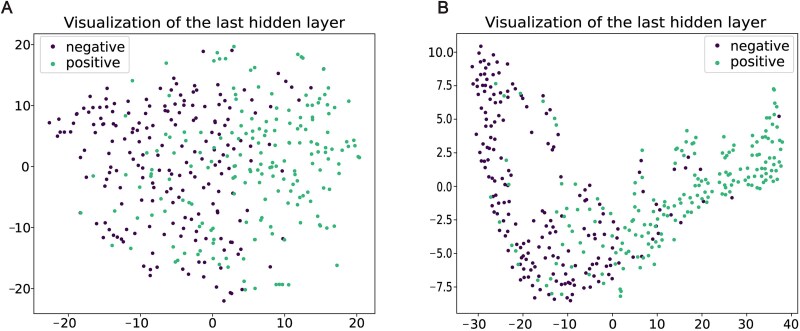
Feature dimensionality reduction visualization analysis. (A) The hidden layer of ProtT5 is visualized by t-SNE dimensionality reduction. (B) The hidden layers of our model are visualized by t-SNE dimensionality reduction.

To more clearly illustrate our model’s ability to learn complex high-dimensional feature representations, we employed the t-SNE method [52] to reduce the multi-dimensional feature space to a 2D plane for visualization, using the test samples as target data. The image reveals the protein embedding features extracted from the ProtT5 model (Fig. 4A). It can be observed that there is some overlap between the positive and negative samples, suggesting that the discrimination between the two classes is not very high in this representation. However, we present the hidden layer feature representations processed by our model, where most of the positive and negative samples are better separated, with only a few samples showing overlap (Fig. 4B). These results strongly validate the effectiveness of our model in learning high-dimensional feature representations of protein sequences.

### Ablation study of model components

To systematically assess the contribution of each module, we conducted an ablation study using five-fold cross-validation. Our proposed model architecture consists of two major components: the ProtT5Encoder and the KmerConvEncoder, with the latter comprising the KmerConv and the MHA sub-modules. We progressively added modules on top of the single backbone to verify their individual contributions, detailed data are presented in Table 4.

**Table 4:** Five-fold cross-validation ablation experimental results (mean ± standard deviation).

Model	Accuracy	Precision	Recall	F1-Score	ROAUC	PRAUC
Baseline	0.772 ± 0.040	**0.794 ± 0.070**	0.704 ± 0.107	0.739 ± 0.038	0.862 ± 0.032	0.864 ± 0.022
+KmerConv only	0.748 ± 0.044	0.786 ± 0.102	0.672 ± 0.175	0.703 ± 0.073	0.859 ± 0.035	0.856 ± 0.016
+MHA only	0.763 ± 0.037	0.739 ± 0.068	0.776 ± 0.080	0.751 ± 0.008	0.867 ± 0.029	0.867 ± 0.019
Full model	**0.776 ± 0.035**	0.756 ± 0.066	**0.780 ± 0.087**	**0.762 ± 0.021**	**0.869 ± 0.033**	**0.868 ± 0.021**

(1) After inserting KmerConv alone, all metrics deteriorated: accuracy dropped by ~2%, recall by ~3%, and F1-score by ~4%. This suggests that the isolated KmerConv injects excessive local noise and constitutes a negative contribution.
(2) When only MHA was appended, precision declined by ~5% while recall increased by 7%, leading to a marginal improvement in overall performance, indicating that the self-attention mechanism systematically shifts the precision–recall trade-off toward higher recall.
(3) Combining KmerConv and MHA boosted recall by ~8% and F1-score by ~2%, while ROAUC and PRAUC remained almost unchanged. This outcome indicates a cooperative effect: KmerConv captures local motifs, MHA establishes global dependencies, and only their joint presence enables the model to exploit both local and global information, delivering optimal F1-score and robust performance.

### Relevant verification

A significant characteristic of proteins undergoing LLPS is the formation of small, spherical, droplet-like structures, often manifesting as regionally concentrated “granules” within the cell [1]. The shape, size, and distribution of these granules can vary depending on the different biomolecules and environmental conditions. Cell Atlas is an image-based subcellular protein distribution map [38], and we searched this map for proteins with high prediction scores. Using the prominent features of PSPs, we identified proteins that are in a condensed or droplet state.

**Figure 5:**
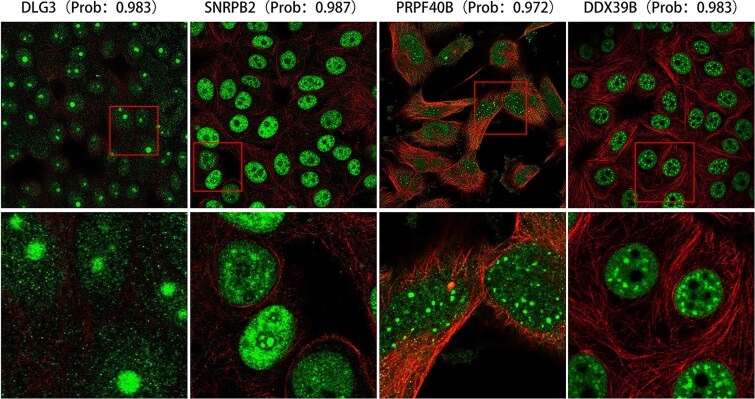
The fluorescence image of the corresponding protein in the HPA database, the upper layer is the original image of the corresponding protein, and the lower layer is the enlarged part of the corresponding red box.

The lower layer image is an enlarged section of the red box from the upper image (Fig. 5). We observed that the protein is in a condensed state and exhibits droplet-like behavior. The fluorescence image of the DLG3 protein, captured in the MCF-7 cell line, exhibits distinct droplet-like structures, with a prediction score of 0.983. Similarly, the fluorescence image of the SNRPB2 protein was also obtained from the MCF-7 cell line. The PRPF40B protein image was captured in the U-251MG cell line, while the fluorescence image of the DDX39B protein was acquired from the MCF-7 cell line. This visual confirmation aligns well with our model’s predictions, demonstrating its effectiveness in identifying PSPs in their droplet states.

**Figure 6:**
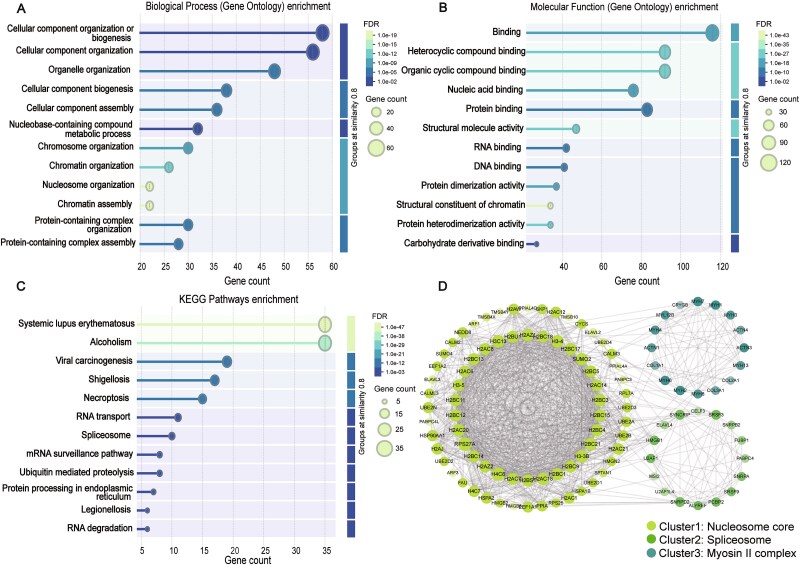
Explore and analyze candidate proteins. (A) GO enrichment analysis (BP) results of candidate proteins. (B) GO enrichment analysis (MF) results of candidate proteins. (C) KEGG enrichment analysis results of candidate proteins. (D) Candidate protein cluster analysis results.

Next, we analyzed the proteins with a prediction score greater than 0.985. After removing the positive data from the training and testing sets, the remaining 128 proteins were considered as candidate proteins. We performed KEGG and GO enrichment analyses on the selected candidate proteins (Fig. 6). Surprisingly, we found that the results were highly consistent with the enrichment analysis of known PSPs.

In the BP enrichment analysis (Fig. 6A), we observed terms such as “Nucleic acid metabolic process” and “Protein-containing complex organization”. In the Molecular Function (MF) enrichment analysis (Fig. 6B), we identified functional terms like “mRNA binding, DNA binding, and RNA binding”. These results suggest that the candidate proteins may play significant roles in nucleic acid metabolism and the organization of protein complexes, and they likely have binding capabilities with mRNA, DNA, and RNA. These characteristics align closely with the known functions of PSPs, implying that our candidate proteins may also be involved in the LLPS process.

Additionally, the KEGG pathway analysis reveals that the candidate proteins are highly enriched in pathways associated with RNA transport, spliceosome, and RNA degradation (Fig. 6C). These pathways are closely related to LLPS proteins, further corroborating findings from previous studies [3]. Furthermore, we observed a notable correlation between the candidate proteins and diseases such as systemic lupus erythematosus (SLE) and alcoholism. Studies have shown that the expression of the USP8 protein is positively correlated with SLE, where it catalyzes the deubiquitination of the SG protein DDX3X, promoting the condensation and activation of cGAS [53]. Therefore, inhibiting USP8 could be an effective strategy for mitigating cGAS-mediated autoimmune diseases. The significant association with alcoholism suggests that these proteins are involved in processes related to neuroadaptation, signal transduction, and gene expression regulation, which is also consistent with previous studies [5].

To further investigate the candidate proteins, we performed a PPI network clustering analysis using the STRING database [48]. Based on the clustering results, the proteins were divided into three clusters (Fig. 6D): nucleosome core-related proteins (Cluster 1, containing 74 proteins), spliceosome-related proteins (Cluster 2, containing 16 proteins), and myosin complex-related proteins (Cluster 3, containing 16 proteins).

Cluster 1 primarily consists of nucleosome core-related proteins. Some of these nucleosome-associated proteins can undergo LLPS to form nucleosomes or participate in nucleosome formation. For example, certain nucleosome proteins have the ability to form or induce LLPS, thereby concentrating rRNA and associated proteins, which aids in nucleosome assembly and function. LLPS can also influence the dynamic properties of nucleosomes, such as nucleosome formation, disassembly, and reassembly [54]. Among the highly connected proteins in this cluster, such as PPIA and EEF1A1, recent studies have shown that PPIA, through its prolyl isomerase activity, can impact protein folding and interactions, thereby regulating LLPS processes [55]. Additionally, PPIA affects the regulation of membrane-less organelles (MLOs) such as stress granules (PABPC1) and P-bodies (DDX6), promoting phase separation and enhancing cellular stress resistance [56]. EEF1A1 interacts with CEP112 and mediates RNA granule assembly through LLPS, controlling post-transcriptional gene expression related to fertility and offering insights into potential therapeutic targets for male infertility [57]. Moreover, proteins such as HSPA1B, RPL7A, and HMGB2 have been shown to participate in the formation of MLOs like nuclear speckles or nucleoli.

Cluster 2 primarily consists of spliceosome-related proteins. The proteins and RNA molecules that make up the spliceosome interact during the splicing process to form a complex 3D structure. Some of these constituent proteins can undergo LLPS to form highly concentrated regions, which can concentrate relevant RNA and proteins, thus enhancing splicing efficiency and promoting spliceosome assembly and function, creating a dynamic and tunable response environment within the cell [5]. According to data from the UniProt database, proteins such as ALYREF, SRSF9, SRSF3, SNRPB2, PABPC4, and U2AF1L4 are involved in the formation of MLOs like nuclear speckles and stress granules. Recent studies have reported that the splicing factor SNRPD2 is upregulated in colorectal cancer, which can interfere with the LLPS of PABPN1, promoting cell proliferation and migration [58]. U2AF1, through its interaction with R-loops and G-quadruplex (G4) DNA structures, can promote LLPS [59], and mutations in U2AF1 directly affect the composition of stress granules [60]. HMGB1 can co-condense with G4s, promoting LLPS through electrostatic interactions [61].

Cluster 3 mainly includes the myosin MYH family and the ACTN family. The MYH family consists of motor proteins that provide energy through ATP hydrolysis to drive intracellular material transport and muscle contraction [62]. ACTN is an actin crosslinking protein that connects actin filaments, forming a network structure that provides structural support and mechanical properties to the cell. The LLPS droplets can concentrate G-actin, increasing its concentration and promoting actin polymerization. Additionally, proteins regulating actin polymerization, such as N-WASP and Arp2/3, can enhance their activity by forming droplet structures with signaling proteins [63].

## Conclusions

Our study establishes a dual-model architecture comprising ProtT5Encoder for universal feature extraction and KmerConvEncoder for fine-grained optimization. Evaluation results demonstrate that ProtT5Encoder supplies foundational embeddings, while the KmerConv and MHA modules act synergistically to exploit both local and global information, yielding robust performance. The unique integrated architecture’s modular design principles allow for task-specific adaptation, and it outperforms standard single-PLMs in evaluations. This approach not only advances our understanding of LLPS mechanisms but also offers novel insights into their potential biological roles and implications in disease. To make it conveniently to study, we made predictions for all human proteins (Supplementary Material S2).

We conducted comprehensive analyses on the candidate proteins, including protein image studies. Imaging validation of high-prediction-score proteins revealed that several proteins exhibited droplet-like aggregates in cell images, consistent with characteristic features of LLPS proteins. Additionally, we performed GO and KEGG pathway enrichment analyses, as well as PPI network analysis, to further investigate functional and interaction characteristics. The results indicated that these proteins share significant similarities with known LLPS proteins, providing additional evidence supporting the robustness of our approach.

While our negative control selection strategy—combining sequence dissimilarity screening with immunofluorescence images verification—improves dataset reliability, critical trade-offs exist: stringent exclusion criteria induce progressive curation bias, which reduces training data diversity while overrepresenting idealized negative specimens. This may compromise model generalizability.

In this study, we focus on sequence-based feature engineering without incorporating auxiliary biological information. Notably, PTMs critically regulate LLPS through biophysical mechanisms. For instance, phosphorylation-induced charge patterning in the IDR of SRRM2 enhances homotypic interaction networks within nuclear speckle molecular assemblies, thereby driving their coalescence [64]. In a follow-up study, we will attempt to introduce PTM features into the model with reference to PTM-Mamba [65].

**Key Points**

- High-quality negative controls were rigorously screened through integrated analysis of sequence dissimilarity and immunofluorescence imaging.
- Feature engineering is performed with protein language models, replacing traditional approaches that rely on expert knowledge.
- A dual-model architecture integrating ProtT5 and KmerConvEncoder was constructed, and ProtT5Encoder supplies foundational embeddings, while the KmerConv and MHA modules act synergistically to exploit both local and global information, yielding robust performance.
- Validated on HPA protein fluorescence images and related literature, and we conducted a series of explorations on candidate proteins.

## Supplementary Material

Supplementary_Material_S1_bbaf681

Supplementary_Material_S2_bbaf681

Supplementary_Material_S3_bbaf681

Supplementary_Material_S4_bbaf681

Supplementary_Material_Table1_bbaf681

## Data Availability

Data can be found at supplemental files and code can be found at https://github.com/xmuzhanglab/PSPsPredict.
