# Supplementary material for: Prediction of liquid–liquid phase separation proteins based on protein language model
Source: Brief Bioinform. 2025 Dec 17;26(6):bbaf681. doi: 10.1093/bib/bbaf681 (PMC12710474; doi:10.1093/bib/bbaf681)
Supplement: Supplementary_Material_Table1_bbaf681 [file supplementary_material_table1_bbaf681.docx]

**Supplementary Table 1 Five-fold cross-validation experimental results (mean ± standard deviation)**

| Model | Accuracy | Precision | Recall | F1-Score | ROAUC | PRAUC | MCC |
| --- | --- | --- | --- | --- | --- | --- | --- |
| PDL | 0.776±0.035 | 0.756±0.066 | 0.780±0.087 | 0.762±0.021 | 0.869±0.033 | 0.868±0.021 | 0.557±0.066 |
| PSL | 0.772±0.040 | 0.794±0.070 | 0.704±0.107 | 0.739±0.038 | 0.862±0.032 | 0.864±0.022 | 0.546±0.070 |
| PLGB | 0.782±0.029 | 0.798±0.031 | 0.710±0.060 | 0.750±0.026 | 0.849±0.029 | 0.854±0.021 | 0.563±0.051 |
| PSVM | 0.791±0.023 | 0.806±0.039 | 0.725±0.041 | 0.762±0.011 | 0.865±0.027 | 0.863±0.018 | 0.581±0.040 |
| PXGBoost | 0.762±0.023 | 0.772±0.039 | 0.687±0.029 | 0.726±0.015 | 0.839±0.027 | 0.846±0.018 | 0.520±0.040 |
| PRF | 0.787±0.028 | 0.830±0.042 | 0.683±0.053 | 0.747±0.023 | 0.847±0.027 | 0.852±0.017 | 0.577±0.045 |
| EDL | 0.764±0.034 | 0.777±0.064 | 0.701±0.084 | 0.731±0.026 | 0.849±0.040 | 0.848±0.020 | 0.529±0.059 |
| ESL | 0.751±0.042 | 0.773±0.060 | 0.673±0.140 | 0.707±0.074 | 0.832±0.041 | 0.834±0.019 | 0.509±0.064 |
| ELGB | 0.749±0.030 | 0.756±0.020 | 0.673±0.044 | 0.712±0.027 | 0.827±0.026 | 0.831±0.010 | 0.493±0.051 |
| ESVM | 0.762±0.046 | 0.763±0.042 | 0.707±0.060 | 0.733±0.037 | 0.823±0.037 | 0.818±0.008 | 0.521±0.086 |
| EXGBoost | 0.758±0.024 | 0.762±0.032 | 0.691±0.041 | 0.724±0.027 | 0.827±0.039 | 0.830±0.024 | 0.513±0.044 |
| ERF | 0.764±0.023 | 0.781±0.020 | 0.679±0.042 | 0.725±0.017 | 0.818±0.026 | 0.820±0.005 | 0.525±0.034 |
